# Molecular mechanisms of lipid droplets-mitochondria coupling in obesity and metabolic syndrome: insights and pharmacological implications

**DOI:** 10.3389/fphys.2024.1491815

**Published:** 2024-11-11

**Authors:** Chunmei Zhang, Mingxuan Zheng, Runlin Bai, Jiale Chen, Hong Yang, Gan Luo

**Affiliations:** ^1^ School of Medical and Life Sciences, Chengdu University of Traditional Chinese Medicine, Chengdu, China; ^2^ Department of Orthopedics, Chengdu Integrated Traditional Chinese Medicine & Western Medicine Hospital/Chengdu First People’s Hospital, Chengdu, China

**Keywords:** lipid droplets, mitochondria, peridroplet mitochondria, obesity, metabolic syndrome, pharmacotherapy

## Abstract

Abnormal lipid accumulation is a fundamental contributor to obesity and metabolic disorders. Lipid droplets (LDs) and mitochondria (MT) serve as organelle chaperones in lipid metabolism and energy balance. LDs play a crucial role in lipid storage and mobilization, working in conjunction with MT to regulate lipid metabolism within the liver, brown adipose tissue, and skeletal muscle, thereby maintaining metabolic homeostasis. The novelty of our review is the comprehensive description of LD and MT interaction mechanisms. We also focus on the current drugs that target this metabolism, which provide novel approaches for obesity and related metabolism disorder treatment.

## 1 Introduction

Obesity has escalated to epidemic proportions worldwide and constitutes a grave threat to public health (Seidell and Halberstadt, 2015). There is also a marked increasing in these conditions among children. The primary cause of obesity is the accumulation of abnormal body fat, which significantly increases the risk of metabolic syndromes that include non-alcoholic fatty liver disease (NAFLD) (Younossi et al., 2016), high blood cholesterol, and diabetes (Kaze et al., 2021). These conditions severely affect health and substantially increase the risk of mortality (Chen et al., 2019). Research into the mechanisms underlying abnormal fat metabolism may be the new perspective for treating obesity and metabolic syndromes.

LDs and MT work cooperatively in lipid metabolism and energy maintenance. LDs-associated MT reveals the existence of various MT groups in cell structures carrying unique protein sets and exhibiting diverse capabilities in fatty acid oxidation (FAO). As mitochondrial motility is a key parameter in mitochondrial fusion, peridroplet mitochondria (PDM) segregate from cytoplasmic mitochondria (CM) due to the distinct mechanisms of mitochondrial adhesion to LDs. Aerobic exercise diminishes the interactions between hepatic LDs and MT, alongside reducing LD size, which correlates with a less severe manifestation of NAFLD (Bórquez et al., 2024). Perilipin 5 (PLIN5) is a lipid droplet-associated protein can regulate lipid metabolism through protein kinase A (PKA) phosphorylation (Gao et al., 2017; Keenan et al., 2021). In this paper, we have summarized the mechanism of LD-MT interaction in abnormal lipid deposition, which might be an influential factor in the etiology of obesity and metabolic syndrome. We also mention the current drugs that target LD-MT interaction which provide novel approaches for obesity treatment.

## 2 Lipid droplets

### 2.1 Structure of lipid droplets

LDs originate in the endoplasmic reticulum (ER) and are associated with general organelles through membrane contact sites. Generally distributed in both prokaryotic and eukaryotic cells (Ibayashi et al., 2024), LDs are lipid and phospholipid storage organelles that serve as centers of lipid metabolism (Zadoorian et al., 2023). Almost all types of cells have the ability to store excess energy in the form of triacylglycerol (TAG) in LDs. LDs store neutral lipids (NLs) and proteins involved in lipid metabolism and cell membrane synthesis, serving as hubs for metabolic processes (Gross and Silver, 2014). LDs consist of a core of NL surrounded by a phospholipid monolayer, which is modified by specific proteins called LDs-associated proteins (LDAP) (Jarc and Petan, 2020), Figure1B.

**FIGURE 1:**
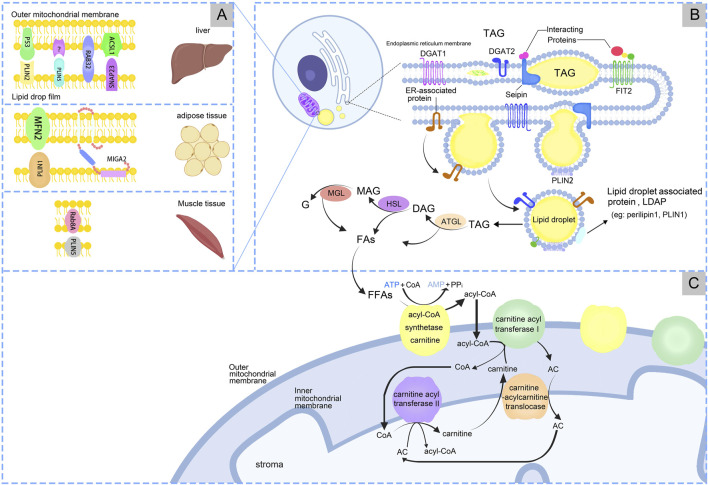
The processes of production and metabolism of lipid droplets. **(A)** Lipid droplets-Mitochondria have receptor mechanisms in different tissues. **(B)** LDs synthesis in endoplasmic reticulum. **(C)** LDs produce free fatty acids (FFAs), which are finally metabolized under the action of mitochondria.

### 2.2 Synthesis and catabolism of lipid droplets

LDs formation is the process of synthesizing and assembling NL and phospholipid monolayers in the ER. This process involves the synthesis of NL within the ER, followed by nucleation, cytoplasmic outgrowth, and growth (Thiam and Ikonen, 2021). The NL stored in LDs are TAG and sterol esters (SE), with TAG being the predominant form. During LDs formation, diacylglycerol acyltransferases (DGAT), including DGAT1 and DGAT2, catalyze the covalent addition of a fatty acyl chain to diacylglycerol, resulting in the esterification and synthesis of TAGs (Wang et al., 2024). TAG is synthesized in the bilayers of the ER, and once a level of 5%–10% is reached, the proteins Fat Storage Inducing Transmembrane Protein 2 (FIT2) and Perilipin3 promote the development of TAG accumulates, LDs precursors (LDP) form “lens-like structures” (Walther et al., 2017). LDP is recruited to the site of LD biogenesis through interactions with LD markers, and at this site LDP bud from the ER into the cytoplasm (Cottier and Schneiter, 2022). LDP originate from the ER and subsequently mature within the cytoplasm (Wang et al., 2016; Demirel-Yalciner et al., 2024), Figure 1B.

The process of lipolysis entails the sequential hydrolysis of TAGs to form diacylglycerols (DAGs) and monoacylglycerols (MAGs), with the liberation of a fatty acids (FAs) at each stage. The final FAs is released upon hydrolysis of the MAG, accompanied by the generation of glycerol (G). Enzymes associated with lipolysis process include adipose triglyceride lipase (ATGL), hormone-sensitive lipase (HSL), and monoacylglycerol lipase/α/β hydrolase domain-6 (MGL/ABHD6) (Ding et al., 2024). Lipophagy mediates the transfer and degradation of triglyceride-containing LDs through the lysosomal pathway (Kaushik and Cuervo, 2015; Wei et al., 2024). The resulting fatty acids undergo β-oxidation in MT. While the regulatory mechanisms of lipophagy remain unclear, this process significantly impacts hepatic metabolic disorders. Further research is needed to elucidate its precise role in liver metabolism (Kaushik and Cuervo, 2015). During the lipolysis process, ATGL breaks down TAG into DAG and FAs. Subsequently, HSL hydrolyzes DAG into MAG and FAs. Finally, MAG is converted into G and FAs by monoacylglycerol lipase (MGL). For a long time, HSL was believed as the rate-limiting enzyme in TAG breakdown. But currently ATGL is the rate-limiting enzyme that catalyzes the first step of TG breakdown to G and FAs (Xie et al., 2024). Mutations in the ATGL gene cause neutral lipid storage disease and myopathy, and reduced ATGL expression has been found in NAFLD (Ghosh et al., 2016). Disruptions in LD metabolism is associated with various metabolic disorders (Carotti et al., 2020), Figure 1B. LDs also response to inflammation, combined with MT, ER, and peroxisomes. These complexes undergo changes in inflammatory stimuli, can supply FAs for LD growth, and support FAs efflux from LDs. Macrophages utilize LDs for inflammatory lipid transport and influence inflammatory lipid mediators, indicating the importance of organelles in the regulation of inflammatory lipid metabolism (Zimmermann et al., 2024). Multi-spectral organelle imaging can comprehensively display the mapping of metabolic organelles including catalase, MT, Golgi apparatus and lysosomes, LD and ER, and their interactions with macrophages. This method can be applied in the study of metabolic changes in macrophages especially lipids which are rapidly responsive, opening up new avenues for potentially targeting the treatment of pathological conditions characterized by dysregulated lipid metabolism.

### 2.3 Lipid droplets associated proteins

LDs proteomics examined more than 200 proteins and functions that collaboratively regulate the droplets’ formation, stability, metabolism, and growth. The aforementioned proteins can be broadly classified into these categories: membrane-associated structures of LDs that protect LDs and regulate their function, e.g., PLIN (Perilipin) and LSD2 (Lysine-Specific Histone Demethylase 2); lipid-metabolising enzymes on the surface of the ER and LDs that synthesise and degrade neutral lipids, e.g., DGAT1 and HSL. Membrane transporter proteins on the surface of the ER and LDs that regulate the interaction of LDs with other cellular structures, e.g., RAB18 (Ras-Related Protein Rab-18, Member RAS Oncogene Family); signalling proteins involved in inflammation and signal transduction, e.g., MAPK (Mitogen-Activated Protein Kinase); and degradation-associated proteins that degrade LD-related proteins, e.g., UBXD8 (UBX Domain-Containing Protein 8) also known as FAF2 (Fas Associated Factor Family Member 2). Furthermore, additional proteins, including ribosomal proteins, histones and actin, are implicated in protein translation, chromosome assembly under stress and LD motility, respectively. Among which perilipins are ubiquitously found in the cytoplasmic LDs of mammalian cells, all play roles in LD function under differing conditions (Cinato et al., 2024). Five genes encode the five main perilipin (PLIN) family proteins (Sztalryd and Brasaemle, 2017), namely, PAT family including Perilipin A (PLIN A), Adipose Differentiation-Related Protein (ADRP), Tail-Interacting Protein 47 (TIP47), Adipocyte Protein S3-12(S3-12), and Oxidative Tissue-Enriched PAT Protein (OXPAT). Their function are various. PAT proteins regulate the lipases into LDs which promote LDs generation. Besides, PAT proteins can also control the lipolysis of stored NL via cytoplasmic lipases, maintain the morphology of LDs, and regulate the movement of LDs (Bickel et al., 2009). PLIN A is a surface protein on LDs that is identical to PLIN 1. PLIN A is predominantly expressed in WAT, and to a lesser extent is present in BAT, cardiac muscle liposarcoma, where its action functions in hormone-induced lipolysis large LD stabilization. PLIN one is a surface protein on LDs that facilitates the stabilization of LDs and lipolysis, which is mediated by lipases and cofactors. The PKA signaling is activated by PLIN one phosphorylation, then inducing the catabolism of TAG. This process has been linked to the development of metabolic diseases such as obesity, diabetes, endocrine disorders, and hypertension (Desgrouas et al., 2024). Furthermore, PLIN one was positively related to disease-free survival (DFS) in lung squamous cell carcinoma (Kim et al., 2023). The depletion of B-cell receptor-associated protein 31 (BAP31) inhibited adipogenesis and lipolysis, but promoted the aberrant enlargement of LDs by reducing the proteasomal degradation of PLIN1 (Wei et al., 2023). ADRP is known as PLIN two that mainly expressed in liver, followed by premature adipocytes, macrophages, sebocytes, mary gland epithelia, choriocaricinoma cells. The functions of PLIN2 include the differentiation of adipocytes, the generation of small LDs, and the stabilization of LDs. ADRP can target adenosine monophosphate-activated protein kinase alpha (AMPKα)-dependent lipophagy, mediated by the Perilipin 2-lysosomal acid lipase (PLIN2-LIPA) axis which ameliorates NAFLD (Fang et al., 2024). ADRP knockout inhibits the induction of platelet-derived growth factor (PDGF) and suppresses vascular smooth muscle cell (VSMC) proliferation in atherosclerotic lesions, as evidenced by a decline in extracellular signal-regulated kinase (ERK) activity and protein kinase B signaling pathways (Zhao et al., 2017). TIP47 is called PLIN three that mainly expressed in ubiquitous but also in skeletal muscle neutrophils, mast cells retinal pigment epithelium sebocytes and its action function is LD stabilization (compensation of PLIN2) PGE2 production intracellular trafficking. PLIN-3 induced apoptosis in CD8^+^ T lymphocytes and promoted Programmed Cell Death 1 Ligand 1 (PD-L1) and B7 Homologue 2 (B7-H2) expression in oral squamous cell carcinoma (OSCC). PLIN-3 knockdown in tumors reduces LDs and tumor migration (He et al., 2024). S3-12 is known as PLIN 4, predominantly expressed in WAT and secondarily occurs in hMSC (Musculin), which is induced during differentiation of skeletal muscle. PLIN four plays a major role in human adipocyte differentiation. Moreover, S3-12 binds to the surface of nascent LDs promoting TAG synthesis in a time-substrate- insulin-dependent manner in LDs generation (Wolins et al., 2003). OXPAT is named of PLIN 5, which is expressed predominantly in cardiac muscle BAT skeletal muscle and to a lesser extent in slet β-cells hepatic stellate cells, where it primarily plays a role in LD stabilisation FA supply to MT. Through augmentation of FAs uptake and elevation of the expression levels of enzymes associated with oxidative catabolism, OXPAT facilitates mitochondrial FAO (Wolins et al., 2006). The heterogeneity of LDAP in different cells and tissues is the result of a combination of factors, including differences in cell type and function, differences in the regulation of protein expression, differences in the intracellular environment, and differences in the interaction of LDs with other organelles. These differences enable lipid droplet proteins to fulfil specific roles in different physiological and pathological states. The proper functioning of LADP is essential for maintaining cellular energy balance and metabolic health. However, disruptions in LADP function are increasingly being recognized as significant contributors to various diseases.

## 3 Mitochondria

### 3.1 The structure of mitochondria

The outer membrane (OM) separates the MT from the cytoplasm While the inner membrane (IM) forms the mitochondrial matrix. The IM is divided into an inner boundary membrane, parallel to the OM, and cristae. Mic60, a component of the MT’s contact site and cristae organizing system, is systematically distributed at cristae junctions to support the mitochondrial structure (Stoldt et al., 2019). Carnitine palmitoyltransferase, a crucial enzyme in lipid β-oxidation, is located on the OM and catalyzes the conversion of palmitoyl CoA to palmitoyl carnitine, a process that is essential for the complete oxidative catabolism of FAs.

### 3.2 Distribution and classification of mitochondria

**FIGURE 2:**
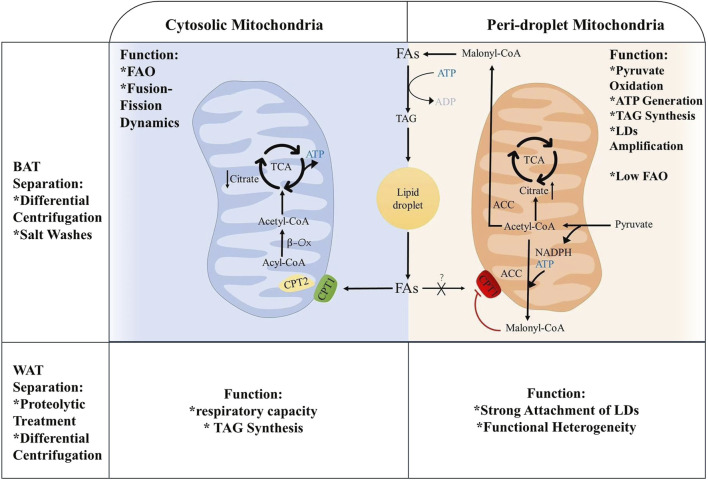
Metabolic differences between periplasmic and cytoplasmic mitochondria.

MT supply energy for the body, while liver, skeletal muscle, cardiovascular and brown adipocytes are the most energetic organs. In LDs metabolism, MT is involved not only in lipogenesis but also in FAO, processes that are intricately linked to mitochondrial dynamics, including movement, fusion, and fission. Microtubule stabilizers induce mitochondrial fusion, activate the mechanistic target of rapamycin (mTOR) signaling pathway and enhance ATP production (Cho et al., 2021; de Mello et al., 2018). Based on the specific proteins of MT and their roles in FAs and pyruvate oxidation, these organelles are categorized into PDM and CM, both of which coexist within the same cell to facilitate lipid oxidation and synthesis (Benador et al., 2018). The functions of PDM and CM differ mainly because the different membrane surface proteins of each play different roles and are closely linked to the interaction with the microenvironment and other organelles. The growth and degradation processes of LDs, the concentration of surrounding FAs and the local redox state are bound to affect PDM (Mahdaviani et al., 2017). In BAT, PDM has stronger pyruvate oxidation, electron transfer and ATP synthesis capabilities. It can also support LD amplification by esterifying FAs into triglycerides with the provision of ATP. CM shows stronger FAO ability (Benador et al., 2018). In WAT, PDM has stronger attachment to LDs through specific protein-protein or protein-lipid interactions, but has lower respiratory and ATP synthesis capabilities than CM. Moreover, the heterogeneity of PDM function can be determined by the size of LDs. The respiratory capacity of PDM is negatively correlated with the size of LDs (Brownstein et al., 2024). The reason for the gap between PDM attached to smaller LDs in WAT having higher respiratory capacity under pyruvate and PDM attached to larger LDs in BAT having higher ATP synthesis ability is that BAT has greater *de novo* lipogenesis and TAG turnover capacity, which is closely related to ATP synthesis and respiration, Figure 2. Naturally, PDM is not exclusive to adipose tissue, and it is likewise found in cardiomyocytes.

### 3.3 Identification and extraction of peridroplet mitochondria

Transmission electron microscopy (TEM) can directly identify PDM, allowing observation of its morphological features (Benador et al., 2018; Freyre et al., 2019; Tarnopolsky et al., 2007), including the arrangement of cristae in PDM towards LDs (Benador et al., 2018; Herms et al., 2015). Confocal imaging microscopy allows real-time observation of the dynamics of LD-MT (Stone et al., 2009; Wang et al., 2011) by double staining LD and MT markers in tissues marker (Benador et al., 2018). Besides, protein blot analysis and silver staining assay are applied to LD-MT mutual studies (Freyre et al., 2019; Cui et al., 2019; Yu et al., 2015), such as detecting PLIN5, mitochondrial proteins, and PLIN2. In addition, proximity ligation assay (PLA) and subcellular fractionation can also assess the status of mitochondria-associated endoplasmic ER membranes (Yang et al., 2019). Differential centrifugation and proteases could successfully separate PDM from LDs (Benador et al., 2018). However, centrifugation alone may not fully eliminate MT from LDs completely, proteases are required for efficient separation (Cui et al., 2019). In WAT, treating PDM with Proteinase K (Prot-K) before differential centrifugation could improve separation efficacy (Brownstein et al., 2024).

## 4 Lipid droplets-mitochondria interaction mechanism

### 4.1 Co-regulation of lipid metabolism by lipid droplets-mitochondria

MT produces ATP and participates in lipid metabolism within LDs. When the cell requires energy, TAG is hydrolyzed to FAs, serving as energy carriers, Figure 1B. FAs are transported intracellularly to various cellular compartments by binding to the cytoplasmic lipid-binding protein, which maintains the solubility of FAs. MT convert FAs to lipoyl-coenzyme A (acyl-CoA) through the carnitine palmitoyltransferase (CPT1/2) system. Firstly, FFAs are catalyzed by acyl-CoA synthase and carnitine acyl transferase I (CPT1) on the OM of MT to form acyl-CoA and acyl-carnitine (AC). Carnitine-acylcarnitine translocase facilitates the transfer AC across the IM into the mitochondrial matrix. AC and CoA are enzymatically reconverted to acyl-CoA by carnitine acyl transferase II (CPT2) on the IM for β-oxidation (Talari et al., 2023), Figure 1C. FFAs are β-oxidized by highly FAO-capable CM to generate acetyl-CoA. Acetyl-CoA serves as a substrate in the tricarboxylic acid cycle (TCA) and oxidative phosphorylation to produce ATP for cells. The translocation of FAs from the LDs to the MT, which necessitates close contact between the two for lipid transfer, reduces FAs exposure and cellular lipotoxicity. In contrast, the enhanced ATP synthesis capacity of PDM facilitates the esterification of FAs to TAG and encourages LDs amplification. Additionally, PDM plays a pivotal role in the conversion of pyruvate to acetyl-CoA, which subsequently undergoes carboxylation by acetyl-CoA carboxylase (ACC) to form malonyl-CoA, an essential precursor for FA biosynthesis (Benador et al., 2018). When pyruvate oxidation through PDM is enhanced, acetyl-CoA levels significantly increase in the mitochondrial matrix. This elevation also impacted malonyl-CoA levels (Benador et al., 2018). At this juncture, malonyl-CoA acts as an inhibitory factor for CPT1, thus effectively blocking the entry of FAs into the MT (McGarry et al., 1978), Figure 2.

### 4.2 The connection of lipid droplets-mitochondria interplay

The interactions between LDs and MT are facilitated by specific contact sites, Table 1. The expression levels and activities of proteins involved in the interaction between LDs and MT exhibit significant heterogeneity across different tissues. This heterogeneity can be attributed to the fact that the gene expression patterns of different tissue cell types are tailored (Elmentaite et al., 2022) to meet the unique energy metabolism and cellular function requirements of their respective tissues (Ouyang et al., 2023; Ferreira et al., 2018). In liver, PLIN5 enhanced LDs formation and increased contacts between LDs and MT (Tan et al., 2019). When high fat diet (HFD), Rab32 localizes to LDs and MT, targeting LDAP-associated proteins Atgl and Plin5 to promote LDs accumulation and inhibition MT biosynthesis, fusion and oxidation. Inhibition of the Creb-Pgc1α pathway blocked Rab32 localization to LDs and MT, suggesting that HFD may target LD-MT and then regulate hepatic LDs lipolysis (Song et al., 2020). Synaptosome-associated protein 23 (SNAP23) and long-chain acyl-CoA synthetase 1 (ACSL1) are situated on the OM of MT, which promote FAs β-oxidation (Che et al., 2023). SNAP23 may be crucial for the LD-MT complex (Jägerström et al., 2009), when ablation would inhibit the complex and β-oxidation. In adipose tissue, mitoguardin 2 (MIGA2) located on OM of MT, facilitate the synthesis of TAG from non-lipid precursors (Freyre et al., 2019). The interaction between PLIN1 and mitofusin 2 (MFN2) also facilitated the association of MT with LDs (Boutant et al., 2017). MT typically accumulate around LDs in adipocytes. Caveolin-1 is involved in LDs and MT interaction, when knockout in adipocytes display the disappearance of MT around LDs and alters the spatial structure between the LDs surface and the cytoplasm, resulting in reduced lipolysis (Cohen et al., 2004). In skeletal muscle, LD-associated PLIN5 established a binding complex with the mitochondrial receptor Rab8a at the surface of LDs, facilitating LDs hydrolysis and delivery of FAs to MT for β-oxidation (Ouyang et al., 2023), Figure 1A.

**TABLE 1:** LD-MT interaction targets between different tissues.

Tissue	Contact site	Model	Result	References
Liver	Acsl1(M), Snap23(L)	Mouse	Promote β-oxidation	Young et al. (2018)
	Acsl1(M)	Mouse	Have a strong linear relationship with weight	Khamoui et al. (2020)
	RAB32 (M, L)	Human hepatocyte cell	Accumulate lipids	Li et al. (2016)
	RAB32 (M, L)	*P. fulvidraco* hepatocytes, human liver cancer cells	Regulating mitochondrial physiological processes	Song et al. (2020)
	P53(M), Plin2(L)	Mouse	Help drive mitochondrial fission	Zhou et al. (2018a)
	Unclear(M) Plin5(L)	Mouse	Promoted LDs Formation	Tan et al. (2019)
Adipose tissue	Miga (M, L)	3T3-L1, COS7 cells	Promotes lipid storage in LDs	Freyre et al. (2019)
	Mfn2(M) Plin1(L)	Mouse	Affected lipolysis	Boutant et al. (2017)
	Caveolin-1(L)	Caveolin-1 null mice	Regulates lipids metabolism	Cohen et al. (2004)
Skeletal muscle	Rab8a(M) Plin5(L)	Rat	Promote FAs oxidation	Ouyang et al. (2023)
	Plin5(L)	LE rats (Male)	Participate in lipolysis	Ramos et al. (2014)

Mshort for located on the Mitochondrial membrane; L: short for located on the LDs, film.

LE, rats: Long-Evans rats.

The contacts between LDs and MT are displayed dynamic and stable, which could not be completely separated during ultracentrifugation. Morphological studies reveal that the interaction between LDs and MT are highly dynamic, consistent with the 'kiss and run’ model. Electron microscope observed that the interaction between LDs and MT in skeletal muscle increases with exercise (Tarnopolsky et al., 2007). Furthermore, there is experimental evidence from electron microscopy that contact between the two varies with experimental conditions (Valm et al., 2017). Despite the publication of a limited number of precise LD-MT interaction protein sites, the molecular biology of how target proteins regulate the binding or separation of the two remains unclear. This represents a significant avenue for future research.

### 4.3 Factors affecting the connection of lipid droplets-mitochondria interplay

The attachment of regulatory proteins associated with LDs and MT interactions represents a complex biological process. In addition to being influenced by the localization and expression of proteins, the spatial distance and nourishment are also significant factors. This section is dedicated to the non-protein factors, and how specific proteins regulate LDs and MT binding and segregation is detailed in subsequent sections.

#### 4.3.1 Spatial distance

MT surrounding LDs were observed in adipocytes, with similar findings in liver cells (Kalashnikova and Fadeeva, 2006). TEM revealed direct contact between LDs and MT. Studies show an association between the size of LDs and their proximity to MT, indicating that larger LDs were more likely to be closely associated with MT, and more efficient FAs transportation. The spatial distance between LDs and MT may affect FAs transportation and lipid metabolism.

#### 4.3.2 Nourishment

Insufficient nutrients activate autophagy to release FAs, which convert to acylcarnitines on the OM of MT by binding to the coenzyme A (COA) moiety. FAs are then transported into the MT matrix via the CPT1/2 system and participate in FAO and oxidative phosphorylation to generate ATP (Nguyen et al., 2017). When overnutrition, overproduced pyruvate, the high pyruvate oxidation capacity in PDM generates substantial malonyl-CoA, which inhibits CPT1 and negatively regulates the transfer of FAs between LDs and MT, thus reducing the rate of lipid metabolism and energy production (McGarry et al., 1978). Conversely, diminished pyruvate oxidation capacity in the CM results in decreased levels of citrate and malonyl-CoA, thereby promoting the translocation of FAs to MT by CPT1 for β-oxidation, Figure 2.

## 5 An overview of lipid droplets-mitochondria interactions in metabolic diseases

### 5.1 Liver tissues

The LD-MT interaction sites known from current studies in the liver are Rab32, ACSL1-SNAP23, p53-PLIN2, and PLIN5. The liver is indispensable in the regulation of lipid metabolism. Rab proteins (Nguyen et al., 2016) are associated with intracellular LDs, and Rab32 is the Rab GTPase associated with MT (Bui et al., 2010). The knockdown of Rab32 promotes lipolysis by indirectly increasing the expression of adipose ATGL (Li et al., 2016). Hepatic FAs significantly upregulate Pgc1α and induce Rab32 localization to LD and MT (Song et al., 2020). NAFLD is one of the most common liver diseases worldwide, characterized by elevated levels of reactive oxygen species (ROS), and Bailey (Bailey et al., 2015) demonstrated that LDs can act as antioxidant organelles. Overexpression of PLIN5 promotes the formation of LDs and LD-MT contacts, reduces cellular levels of ROS and upregulates genes related to mitochondrial function. This represents a survival strategy adopted by cells in response to stress and is a promising new target (Tan et al., 2019). Moreover, oleic acid (OA) increases PLIN5 via the PI3K/PPAR(Peroxisome Proliferator-Activated Receptor) α pathway (Zhong et al., 2019), and IL-6 increases PLIN5 through the JAK/STAT3 axis (Krizanac et al., 2023). The increase in PLIN5 promotes the contact between LD and MT, reduces the cellular levels of ROS. In contrast, Chaperone-mediated autophagy (CMA) induces Plin5 degradation (Ma et al., 2020). In addition, lipid metabolism defects not only cause NAFLD but also insulin resistance (IR). Abnormal lipid metabolism in hepatocytes leads to increased ROS. These responses in turn affect insulin signalling in the liver, resulting in decreased liver sensitivity to insulin. This leads to a reduction in hepatic uptake and utilisation of glucose and exacerbates IR (Tilg et al., 2021). The livers of Plin5-deficient mice exhibited activation of c-Jun N-terminal kinase, impaired insulin signal transduction, and IR, which impaired systemic insulin action and glycemic control. The re-expression of Plin5 reversed these effects (Keenan et al., 2019). Similarly, 17β-HSD13 functions as a hepatocyte-specific LDAP, and its expression is upregulated in patients with NAFLD (s). Besides, comparative proteomic study reveals 17β-HSD13 as a pathogenic protein in NAFLD (Su et al., 2014). Inadequate dietary choline intake is associated with choline deficiency (CD), which induces NAFLD (Michel et al., 2011). Knockdown of serine/threonine kinase (STK) on the surface of hepatic LDs increases lipolysis and protects hepatocytes (Kim et al., 2023). Another new entry point for the treatment of NAFLD is the NR4A1 (nuclear receptor subfamily four group A member 1)/DNA-PKcs (DNA-dependent protein kinase catalytic subunit)/p53 pathway. The regulation of p53 is bidirectional, driving MT fission on the one hand, and stalling MT autophagy on the other to maintain MT homeostasis. Furthermore, melatonin blocks the NR4A1/DNA-PKcs/p53 pathway, promotes mitochondrial autophagy, and enhances NAFLD (Zhou et al., 2018a). ACSL1 is situated in the mitochondrial OM. The direct interaction with CPT - one leads FAs from LDs to the MT for oxidation, while the tethering of SNAP23 is not required (Young et al., 2018). The liver injury results in increased energy expenditure and weight loss, with ACSL1 content being linearly correlated to body weight (Khamoui et al., 2020). Elevated ACSL1 expression decreases β-oxidation (Li et al., 2020). Furthermore, miR-34c promotes liver fibrosis by inhibiting ACSL1 expression (Li et al., 2021). PLIN2 deficiency attenuates hepatic steatosis. The lack of Cannabinoid receptor 1 (CB1) signaling results in a decrease in PLIN2 levels through the CB1-perilipin2 axis, which opens up ideas for the treatment of steatosis (Irungbam et al., 2020). In alcoholic fatty liver disease (AFLD), p53 interacts directly with ALDH2, inhibiting the formation of reactive tetramers and indirectly limiting pyruvate production (Yao et al., 2023). PLIN2 inhibits the adenosine 5′-AMPK/ULK1 (human) recombinant protein-lysosome pathway and promotes cell proliferation in hepatocellular carcinoma (HCC) (Liu et al., 2022). Additionally, RAB32 is a crucial target of miR - 30c - 5p in HCC. miR - 30c - 5p inhibits the growth and invasion of HCC cells via the miR - 30c - 5p - RAB32 axis (He et al., 2021).

### 5.2 Brown adipocytes

The LD-MT sites are mainly MIGA2, PLIN1-MFN2. High MIGA2 expression not only promotes LD-MT contact but also effectively activates adipocytes to synthesize TAG from non-lipid precursors (Freyre et al., 2019). The direct interaction between Mfn2 and PLIN1 is conducive to the entry of FAs into MT and FAO. Mfn2 knockout would impair the fat utilization in mice even under a low-fat diet (LFD), increasing the incidence of obesity (Boutant et al., 2017).

### 5.3 Skeletal muscle

Skeletal muscle activity requires a large supply of mitochondrial energy, and the known LD-MT sites are PLIN5-Rab8A and PLIN2. PLIN5 mediates LD-MT coupling (LDMC) to enhance mitochondrial respiratory capacity, and its abundance correlates with mitochondrial respiration rates (Bosma et al., 2012; Kien et al., 2022). Vitamin D upregulates PLIN2 levels. In myotubes, calcitriol (the active form of vitamin D) increases the mRNA of triglyceride synthesis genes DGAT1 and DGAT2, partially mediated by PLIN2 for mitochondrial oxidative function (Schnell et al., 2019). Interestingly, overfeeding upregulates PLIN2 expression, but has no effect on ROS release *in vivo* (Toledo et al., 2018). Vascular endothelial growth factor B (VEGFB) upregulates fatty acid transporter protein 4 (FATP4) and FATP1, inhibiting FASN production (Li et al., 2019). Caffeine promotes FAs utilization and FAO (Enyart et al., 2020).

### 5.4 Cardiovascular

Myocardial disease is the most common cause of mortality and disability globally (Christiansen et al., 2017; Wende et al., 2017). A certain degree of LDs can alleviate oxidative stress in cells (Jarc and Petan, 2019). Oxidative stress is an imbalance between ROS and antioxidants in cells (Burgoyne et al., 2012). Under physiological conditions, ROS regulates numerous cellular processes at low concentrations (Lennicke and Cochemé, 2021); however, overproduction of ROS results in impaired cellular components and function (Ong et al., 2015; Humeres and Frangogiannis, 2019), triggering adverse cardiac remodeling and progression to heart failure (De Geest and Mishra, 2022; D'Oria et al., 2020). ROS induces LDs accumulation by increasing PLIN2 expression, and PLIN2 modulates LDs formation via PPAR and CREBBP (CREB Binding Protein) signaling pathways (Jin et al., 2018a). In mouse hearts, Plin2 is upregulated during fasting-induced steatosis (Suzuki et al., 2009; Ueno et al., 2017). Moreover, Sato demonstrated that Plin2-induced cardiac steatosis leads to an increased incidence of atrial fibrillation in aged mice (Sato et al., 2019). PLIN5 expression inhibits ROS (Zheng et al., 2017). LDs prevent excess ROS production by decreasing FAO (Kuramoto et al., 2012). Plin5 knockout mice exhibit significantly reduced TAG accumulation in cardiomyocytes (Drevinge et al., 2016), increased cardiac hypertrophy, and elevated myocardial oxidative stress following transaortic constriction (Wang et al., 2019). Thus, Plin5-deficient myocardium elevates levels of ROS (Zheng et al., 2017), suggesting that Plin5 deficiency reduces cardiac function.

## 6 Pharmacology research of lipid droplets-mitochondria - Related targets

There are few drug studies showing intervention at the LD-MT interaction site, and to better provide new insights into the treatment and prevention of obesity, we have collected a wide range of drug studies related to LD synthetic catabolism and LD-MT interactions.

### 6.1 Lipid droplets synthesis and catabolism

PPAR-γ, SREBP-1 and C/EBPα/β targets such as FASN, HSL, ATGL, fatty acid binding protein (FABP) and LDAP regulating LDs synthesis and catabolism. PPARs promote LDs accumulation in the liver. Octyl gallate (OG) (Lima et al., 2020) and p-coumaric acid (p-CA) (Yuan et al., 2023) upregulate PPAR-γ expression. Ochratoxin A (OTA) upregulates PPAR γ levels, and induces hepatic steatosis through the PPAR γ-CD36 axis (Zheng et al., 2021). Similarly, the extract from Syzygium simile leaves (SSLE) decreases CD36 expression, hindering cellular LDs accumulation (Yen et al., 2018). Additionally, p-AMPK reduces LD accumulation. The TF3-PK-AMPK regulatory axis is a novel mechanism to alleviate lipid deposition. The theaflavin monomer theaflavin-3,3′-digallate (TF3) acts on the TF3-PK-AMPK regulatory axis and activates AMPK (Zhang et al., 2020a). Conversely, DHA inhibits AMPK phosphorylation (Xia et al., 2021). Additionally, oroxylin A inhibits HIF-1α expression *in vivo*, suppressing LDs accumulation (Jin et al., 2018b). Furthermore, Quercetin induces NAFLD by inhibiting AKT via the PI3K/AKT pathway and promoting FAs synthesis (Li et al., 2023a).

In skeletal muscle, vitamin D reduced PPAR-γ levels *in vivo*, subsequently lowering PLIN2 expression and decreasing LDs in skeletal muscle (Li et al., 2018). Glucagon-like peptide-1 receptor agonists (GLP-1RA) and semaglutide enhance the Sirtuin1 (SIRT1) signaling pathway, leading to a downregulation of the atrophy-associated factor Atrogin-1 and an increase in myogenic factor expression, which alleviating muscle atrophy and enhancing IR (Xiang et al., 2023).

Also in adipocytes, Polysaccharide CM1 diminishes PPAR-γ, DGAT1, and DGAT2 (Yu et al., 2021). Lemon extract (LE) (Carota et al., 2021) and triterpenoid cycloastragenol (CAG) (Kim et al., 2024) downregulate PPAR-γ in 3T3-L1 cells. Curcumin and Synthetic Curcumin Derivatives also downregulate PPAR-γ, suppress COX2, inhibit FASN (Moetlediwa et al., 2024). Mulberry and Hippophae-based solid beverages inhibit TGF-β and PPAR-γ signaling pathways, restoring WAT dysfunction (Zhou et al., 2024). Polychlorinated biphenyls (PCBs) upregulate PPAR-γ and induce fat-specific protein 27 (Fsp27) expression, resulting in IR (Kim et al., 2017). Other pathways that modulated the increase in adipocyte LDs included clozapine, which significantly and directly reduced leptin secretion in 3T3-L1 adipocytes (Tsubai et al., 2017). In fascia-derived stromal cells (FSC), suramin significantly increase PPAR-γ, consequently elevating the expression of PLIN2, causing LDs accumulation (Li et al., 2023b). Rutin directly activates the SIRT1/PGC-1α/mitochondrial transcription factor (Tfam) signaling pathway increasing MT and UCP1 in BAT, ultimately enhancing energy expenditure (Yuan et al., 2017). Olanzapine can increase PLIN1, PLIN2 and PLIN4 expression (Nimura et al., 2015). Deoxyschizandrin (DS) and DS-liposomes (DS-liposomes) in 3T3-L1 adipocytes reduce LDL in the cytoplasm, alleviating NAFLD (Liu et al., 2018).

### 6.2 Drugs associated with lipid droplets-mitochondria

#### 6.2.1 Drugs targeted on the lipid droplets-mitochondria connection

Drugs have the potential to directly target LD-MT interaction proteins to modify the oxidative catabolic process of LDs, aiming to intervene in obesity. Statins reduce hepatocyte TAG content by inhibiting PLIN5 expression (Langhi et al., 2014) Atorvastatin ameliorates NAFLD by enhancing PLIN5 phosphorylation, and reducing TAG accumulation in the liver (Gao et al., 2017). Additionally, glycocoumarin (GCM) regulates the PLIN5-Sirt1 axis, alleviating hepatic lipotoxicity (Zhang et al., 2020b). Resveratrol (RES) inhibits the thioacetamide (TAA)-induced TNF-alpha (inflammatory)/NF-kB (nuclear factor-kappa B)/iNOS (nitrosative stress)/HIF-1α axis (Ebrahim et al., 2022), and ameliorates hepatic steatosis (Zhou et al., 2018b). Tumor suppressor protein p53 reduces LDs (Borude et al., 2018).

#### 6.2.2 Drugs targeted on lipid droplets-mitochondria-related axes

Drugs affect relevant LD-MT metabolites, which modulate lipid metabolism and influence obesity. Among them, CPT1/2, PGC-1α (PPAR coactivator), and AMPK on the MT membrane play a central role. PGC-1α serves as a transcriptional co-activator whose upregulation increases FAO. Pioglitazone upregulates PGC-1α and ameliorates IR (Tan et al., 2020). Conversely, Simvastatin disrupts the Akt/mTOR pathway, hampered insulin receptor and mTORC2 function, inducing IR (Sanvee et al., 2019; Bonifacio et al., 2015). Interestingly, vincristine impairs glycogen muscle reserve in normal mice but not in PGC-1α overexpressing mice, suggesting a role for PGC-1α in preventing simvastatin-associated myotoxicity (Panajatovic et al., 2021; Panajatovic et al., 2020). In addition, dagliflozin treatment for 5 weeks increased CPT1 (Op den Kamp et al., 2022), Table 2.

**TABLE 2:** A meta-collection of drugs acting on LD-MT.

Name	Organ/tissue	Site of action	Result	References
Targeting LDs synthesis and catabolism				
Octyl gallate	Liver	Ppar-γ	Increase in LDs	Lima et al. (2020)
Ochratoxin A	Liver	Ppar-γ and Siah2	Increase in LDs	Zheng et al. (2021)
Syzygium simile leaves	Liver	CD36	Decrease in LDs	Yen et al. (2018)
Theaflavin-3,3′-digallate	Liver	TF3-PK-AMPK axis	Decrease in LDs	Zhang et al. (2020a)
Dihydroartemisinin	Liver	lncRNA-H19 and AMPK	Increase in LDs	Xia et al. (2021)
Oroxylin A	Liver	Hif-1α	Decrease in LDs	Jin et al. (2018a)
Quercetin	Liver	PI3K/AKT (PKB)/mTOR	Decrease in LDs	Li et al. (2023a)
Vitamin D	Skeletal muscle	Plin2	Decrease in LDs	Li et al. (2018)
Glucagon like peptide-1 receptor agonists	Skeletal muscle	Sirt1 and Atrogin-1	Improve insulin resistance	Xiang et al. (2023)
Semaglutide	Skeletal muscle	Sirt1 and Atrogin-1	Improve insulin resistance	Xiang et al. (2023)
Polysaccharide CM1	Adipose tissue	Ppar-γ	Decrease in LDs	Yu et al. (2021)
Cycloastragenol	Adipose tissue	Ppar-γ	Decrease in LDs	Kim et al. (2024)
Lemon extract	Adipose tissue	Ppar-γ and Dgat-1mRNA	Decrease in LDs	Carota et al. (2021)
Polychlorinated biphenyls	Adipose tissue	Ppar-γ	Increase in LDs	Kim et al. (2017)
MHC	White adipose tissue	Ppar-γ and Fgfr1	Decrease in LDs	Zhou et al. (2024)
Curcumin and Synthetic Derivatives	unclear	PPAR-γ、COX2、FAS	Decrease in LDs	Moetlediwa et al. (2024)
Clozapine	Adipose tissue	Leptin	Increase in LDs	Tsubai et al. (2017)
Suramin	Adipose tissue	Srebp1, C/Ebpα, C/Ebpβ and Ppar-γ	Increase in LDs	Li et al. (2023b)
Rutin	Adipose tissue	Sirt1/Pgg-1α/Tfam	Decrease in LDs	Yuan et al. (2017)
Olanzapine	Adipose tissue	Plin1, Plin2 and Plin4	Increase in LDs	Nimura et al. (2015)
Deoxyschizandrin	Adipose tissue	Liposome	Decrease in LDs	Liu (Liu et al., 2018)
Targeting the LD-MT physical linkage site				
Statins	Liver	Plin5	Reduce triglycerides in liver cells	Yuan et al. (2017)
Atorvastatin	Liver	Plin5	Promote PLIN5 phosphorylation to Promote fat breakdown	Bórquez et al. (2024)
Mammalian target of rapamycin 1	Liver	Rab32	Regulates lysosomal-mTOR transport	Liu et al. (2018)
Glycycoumarin	Liver	Plin5-Sirt1 axis	Relieve lipotoxicity	Langhi et al. (2014)
Resveratrol	Liver	Tnf-a/Nf-kb/i Nos/Hif-1a axis	Protect the liver; Reduce accumulation of LDs	Ebrahim et al. (2022)
Paracetamol	Liver	p53	High doses cause acute liver failure	Zhang et al. (2020a)
Targeting the LD-MT-related metabolic axis				
Pioglitazone	Skeletal muscle	Ampk	Decrease in LDs	Tan et al. (2020)
Simvastatin	Skeletal muscle	Akt/mTOR	Reduce glucose transport	Sanvee et al. (2019), Bonifacio et al. (2015)
Simvastatin(PGC-1α is overexpressed)	Skeletal muscle	Fatp4	Increase fatty acid transport	Panajatovic et al. (2021), Panajatovic et al. (2020)
Dapagliflozin	Skeletal muscle	SGLT2	Increase LDs, increase FAO	Op den Kamp et al. (2022)

## 7 Conclusions and perspectives

In lipid metabolism, LDs are involved in membrane synthesis and store neutral lipids and proteins, through interactions with membrane-contact sites in various organelles (Zadoorian et al., 2023; Gross and Silver, 2014). It also regulated functions such as lipase entry into the LDs and maintenance of LDs morphology and motility with the assistance of the LDAP protein family (Bickel et al., 2009). The pyruvate oxidation capacity of PDM in WAT was significantly greater than that of CM (Benador et al., 2018). However, the mechanism behind this enhanced pyruvate oxidation capacity remains unknown. Moreover, PDM and CM have been individually researched in BAT and WAT, but the interconnections need to go deep.

There is substantial scientific evidence to suggest that LD-MT interplay is of great significance to human health (Fan and Tan, 2024). Despite there is a large number of data on LD-MT interplay, the molecular composition and regulation of LD-MT tethering is still unknown. Consequently, future research endeavors should prioritize the identification of novel interacting proteins. LD-MT interacts on the surfaces of LDs and MT with surface proteins, e.g., PLIN5 playing a significant role. Future research can continue exploring contact proteins that interact with PLIN5 in the liver, aiming to enhance understanding of the underlying mechanisms in studies centered on the interplay between LDs and MT.

Medications that target the LDs sites to regulate cellular lipid synthesis and lipolysis. Regarding LD-MT interaction sites, medications can directly impact the oxidative catabolic process of LDs. For instance, statins reduce hepatocyte TAG content by inhibiting PLIN5 expression (Langhi et al., 2014). There is limited research on drugs targeting the LD-MT target, and additional studies are necessary to investigate whether other drugs that promote fat reduction and metabolic enhancement inhibit or reduce body weight through this mechanism. We anticipate the development of potent and side-effect-free medications targeting alternative pathways for treating obesity-related conditions. These advancements will offer novel approaches for managing metabolic disorders associated with obesity.
